# The key features of SARS-CoV-2 leader and NSP1 required for viral escape of NSP1-mediated repression

**DOI:** 10.1261/rna.079086.121

**Published:** 2022-05

**Authors:** Lucija Bujanic, Olga Shevchuk, Nicolai von Kügelgen, Anna Kalinina, Katarzyna Ludwik, David Koppstein, Nadja Zerna, Albert Sickmann, Marina Chekulaeva

**Affiliations:** 1 Non-coding RNAs and mechanisms of cytoplasmic gene regulation, Berlin Institute for Medical Systems Biology, Max Delbrück Center for Molecular Medicine, 10115 Berlin, Germany; 2 Leibniz-Institut für Analytische Wissenschaften—ISAS, 44139 Dortmund, Germany; 3 Systems Biology of Gene Regulatory Elements, Berlin Institute for Medical Systems Biology, Max Delbrück Center for Molecular Medicine, 10115 Berlin, Germany

**Keywords:** SARS-CoV-2, translational repression

## Abstract

SARS-CoV-2, responsible for the ongoing global pandemic, must overcome a conundrum faced by all viruses. To achieve its own replication and spread, it simultaneously depends on and subverts cellular mechanisms. At the early stage of infection, SARS-CoV-2 expresses the viral nonstructural protein 1 (NSP1), which inhibits host translation by blocking the mRNA entry tunnel on the ribosome; this interferes with the binding of cellular mRNAs to the ribosome. Viral mRNAs, on the other hand, overcome this blockade. We show that NSP1 enhances expression of mRNAs containing the SARS-CoV-2 leader. The first stem–loop (SL1) in the viral leader is both necessary and sufficient for this enhancement mechanism. Our analysis pinpoints specific residues within SL1 (three cytosine residues at the positions 15, 19, and 20) and another within NSP1 (R124), which are required for viral evasion, and thus might present promising drug targets. We target SL1 with the antisense oligo (ASO) to efficiently and specifically down-regulate SARS-CoV-2 mRNA. Additionally, we carried out analysis of a functional interactome of NSP1 using BioID and identified components of antiviral defense pathways. Our analysis therefore suggests a mechanism by which NSP1 inhibits the expression of host genes while enhancing that of viral RNA. This analysis helps reconcile conflicting reports in the literature regarding the mechanisms by which the virus avoids NSP1 silencing.

## INTRODUCTION

SARS-CoV-2, responsible for the current global pandemic, manages to evade mechanisms of host immunity during infections to promote its own replication and spread. To achieve this, it simultaneously suppresses the translation of cellular proteins and promotes that of its own, although the two processes require the same basic machinery. Exposing the mechanisms by which it manages this would likely provide insights into crucial stages in the viral lifecycle that might be exploited in therapies.

**FIGURE 1.**
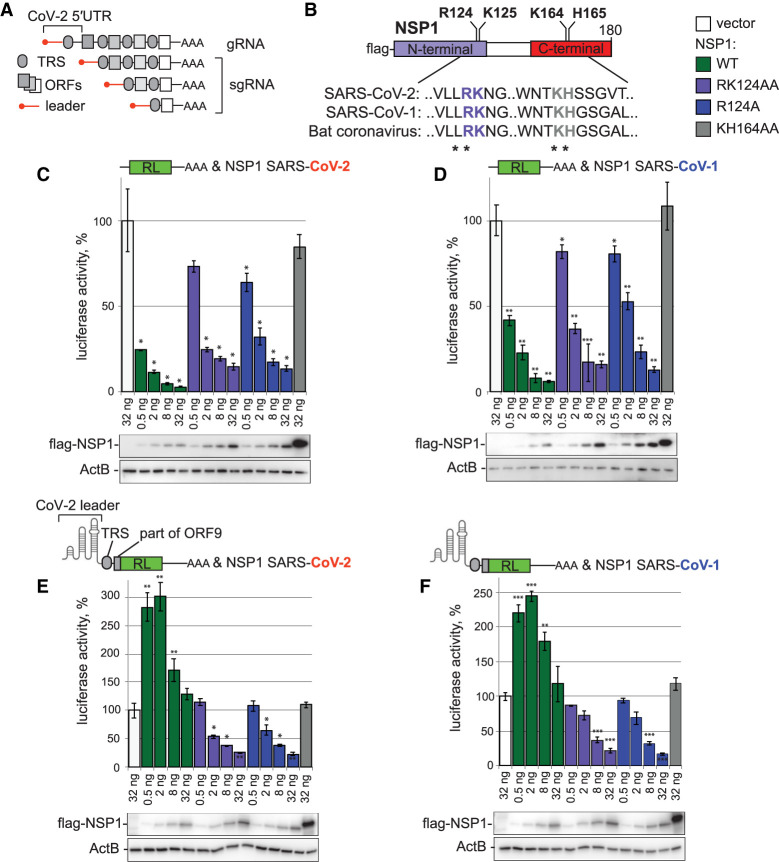
SARS-COV-2 leader enables the virus to escape repression by the NSP1 protein, but not its R124A and RK124AA mutants. (*A*) Schematic representation of genomic SARS-CoV-2 RNA (gRNA) and subgenomic RNAs (sgRNAs). Red line: viral leader; gray oval: transcriptional regulatory sequences (TRS); gray rectangles: open reading frames (ORFs). Please note that not all TRSs and ORFs are shown. (*B*) Schematic representation of NSP1 protein, including analyzed mutants R124A, RK124AA, KH164AA with partial alignment of mutated regions. The numbers correspond to the amino acid positions. (*C*) Repression of RL mRNA by SARS-CoV-2 NSP1 and its point mutants. HEK293T cells were cotransfected with Renilla luciferase (RL) plasmid and increasing amounts of plasmids, encoding flag-tagged SARS-CoV-2 NSP1, or indicated NSP1 point mutants. As negative control, the vector encoding flag alone instead of flag-NSP1 plasmid was used. Open bars: vector, green: WT NSP1, purple: RK124AA NSP1, blue: R124A NSP1, gray: KH164AA NSP1. Values are presented as a percentage of luciferase produced in the presence of the vector. Values represent means ±SD from at least three experiments. *P*-values ([*] <0.1, [**] <0.01, [***] <0.001), calculated with a two-sample *t*-test comparing vector and NSP1 samples, are shown *above* the relevant bars. Expression of flag-NSP1 fusion protein and its point mutants was estimated by western blotting with anti-flag antibodies and shown *below* the reporter assay. Note that KH164AA mutant migrates slower than WT. ActB was used as a loading control. (*D*) Repression of RL mRNA by SARS-CoV NSP1 and its point mutants. The experiment was performed as in *C*, but SARS-CoV NSP1 instead of SARS-CoV-2 NSP1 was used. (*E*,*F*) Reporter bearing SARS-CoV-2 leader (CoV-2-RL) escapes repression by WT NSP1, but is repressed by NSP1 R124A and NSP1 RK124AA mutants. Panel *E* shows the effects of NSP1 from SARS-CoV-2 and panel *F*—NSP1 from SARS-CoV. CoV-2-RL reporter bears the viral leader, TRS and the beginning of viral ORF9 (encoding nucleocapsid protein) fused with the RL coding sequence. See Materials and Methods for more details.

SARS-CoV-2 belongs to the genus beta-coronavirus (Zhou et al. 2020), which also includes a bat coronavirus (96% identity at the genome level with SARS-CoV-2), SARS-CoV-1 (or SARS-CoV), which caused a SARS epidemic in 2003, and Middle East respiratory syndrome (MERS) CoV, responsible for an outbreak of respiratory disease in 2012 (for reviews, see Chen et al. 2020; Yang et al. 2020). The genome of SARS-CoV-2 is a ∼30 kb positive-stranded RNA with 5′-cap structure, 5′UTR, 3′UTR, and poly(A) tail. It contains 10 protein-coding open reading frames (ORFs). Upon cell entry, the genomic RNA (gRNA) is translated into polyprotein, which is processed into 16 nonstructural proteins (NSPs). Subsequently, its gRNA serves as a template to generate a set of subgenomic mRNAs (sgRNAs) that encode other viral proteins. Curiously, all sgRNAs possess a common “leader” sequence, corresponding to a specific region of 70 nt found at the 5′ end of the gRNA. This leader is added to sgRNAs because viral transcription involves template switching at specific regions, called transcriptional regulatory sequences (TRS, Fig. 1A).

The first protein produced by coronaviruses upon infection is NSP1, encoded by ORF1a at the 5′ end of gRNA. NSP1 is an important virulence factor that plays a crucial role in its pathogenicity by helping the virus evade the host innate immune response (for review, see Chen et al. 2020). In the related virus SARS-CoV-1, NSP1 inhibits immunity via two mechanisms: by repressing expression of host transcripts (Kamitani et al. 2006, 2009; Narayanan et al. 2008; Huang et al. 2011; Lokugamage et al. 2012; Tanaka et al. 2012) and by preventing full induction of interferon (IFN) and decreasing STAT1 phosphorylation (Wathelet et al. 2007).

A number of studies have been devoted to determining how SARS-CoV-1 NSP1 represses host gene expression, but some of their conclusions seem contradictory (Kamitani et al. 2006, 2009; Narayanan et al. 2008; Huang et al. 2011; Lokugamage et al. 2012; Tanaka et al. 2012). Two repression mechanisms have been reported: translational repression and mRNA degradation (Kamitani et al. 2009; Huang et al. 2011; Lokugamage et al. 2012). Specific amino acid residues important for NSP1-mediated repression have been identified. NSP1 carrying [K164A; H165A] mutations is fully nonfunctional (Lokugamage et al. 2012; Tanaka et al. 2012), and another [R124A; K125A] mutant lacks the mRNA destabilization function (Lokugamage et al. 2012). The mechanism by which SARS-CoV-1 NSP1 achieves translational repression is not fully understood. Its effects are thought to relate to the general translation machinery, due to its cosedimentation with the small ribosomal subunit (40S) and coimmunoprecipitation with the ribosomal protein S6 (Kamitani et al. 2009). Yet experiments with the separation of translation complexes on sucrose density gradients and toeprinting analyses produced conflicting data. While the former suggested that NSP1 inhibits recruitment of the large ribosomal subunit and formation of the 80S initiation complex, the latter indicated that it rather affected the recruitment of the small (40S) ribosomal subunit and assembly of the 48S initiation complex (Kamitani et al. 2009). Additionally, some reports suggested that SARS-CoV-1 NSP1 affects only host mRNA, while SARS-CoV-1 mRNAs are protected from translational down-regulation through interactions between virus-specific leader sequences with NSP1 (Tanaka et al. 2012). Other studies suggested that viral mRNAs are also translationally inhibited by NSP1 in SARS-CoV-1-infected cells, providing an overall picture that is confusing (Lokugamage et al. 2012; Narayanan et al. 2015).

The COVID-19 pandemic has triggered intensive research into the mechanisms of NSP1 functions in SARS-CoV-2 (Banerjee et al. 2020; Schubert et al. 2020; Shi et al. 2020; Thoms et al. 2020; Tidu et al. 2020; Lapointe et al. 2021; Mendez et al. 2021). Three cryo-EM studies showed that SARS-CoV-2 NSP1 binds to the ribosomal 40S subunit and blocks the mRNA entry tunnel (Schubert et al. 2020; Thoms et al. 2020; Yuan et al. 2020). However, in order for the virus to propagate, viral translation has to proceed in the presence of NSP1. Here, too, attempts to resolve the underlying mechanisms have produced conflicting results. As with studies on SARS-CoV-1 NSP1, some work has reported that SARS-CoV-2 NSP1 represses both host mRNAs and mRNAs with the viral leader (Schubert et al. 2020); other studies found that viral reporters escape repression by NSP1 (Banerjee et al. 2020; Tidu et al. 2020; Mendez et al. 2021). When the evasion of viral reporters from NSP1-mediated repression has been reported, authors have disagreed about the viral elements that are required. Shi et al. (2020) reported that multiple elements in the viral leader make contributions, while Tidu et al. (2020) and Banerjee et al. (2020) argue that a specific stem–loop structure in the viral leader suffices.

Here we use a combination of reporter assays, mutagenesis and mass spectrometry to dissect the mechanisms of SARS-CoV-2 NSP1 function and provide insights into how the virus evades NSP1 silencing. We show that SARS-CoV-2 NSP1 both down-regulates global protein production and fosters the expression of viral reporters. We find that the stem–loop 1 (SL1) in the viral leader is both necessary and sufficient for up-regulation of viral reporters. We map three specific cytosine residues (3C) within SL1 and an arginine residue at the position 124 in NSP1 which are absolutely required for viral evasion. Mutation of any of these four residues, alone or in combination, is sufficient to make the viral reporter susceptible to NSP1 repression. Moreover, we use BioID (Roux et al. 2012) to determine the functional interactome of SARS-CoV-2 NSP1, identifying multiple components of the antiviral defense system.

## RESULTS

### Stem–loop 1 (SL1) is both necessary and sufficient for NSP1-mediated up-regulation of viral RNA expression

To recapitulate SARS-CoV-2 NSP1-mediated repression in HEK293T cells, we set up a luciferase reporter assay. We coexpressed Renilla luciferase mRNA (RL) with NSP1-encoding constructs (Fig. 1B). As negative controls, we used an empty vector and a NSP1 KH164AA [K164A; H165A] mutant reported as nonfunctional due to a disruption of interactions with the ribosome (Schubert et al. 2020; Thoms et al. 2020; Lapointe et al. 2021). As expected, WT NSP1, but not its KH164AA mutant, efficiently repressed luciferase expression in a dose-dependent manner (four- to 30-fold, Fig. 1C; Supplemental Fig. S1A). Because RK124AA [R124A; K125A] and R124A NSP1 mutants have been reported to be partially nonfunctional in previous studies on SARS-CoV-1 (Lokugamage et al. 2012; Tanaka et al. 2012), we also included RK124AA and R124A mutants in the analysis. Both mutants were able to repress mRNA expression, although to a lesser degree than WT (1.5- to sevenfold, Fig. 1C). Similar behavior was observed for SARS-CoV-1 NSP1 and its mutants (Fig. 1D). Consistent with a role of NSP1 in global translational repression, the expression levels of WT NSP1 and its mutants anticorrelated with their strength as translational repressors: WT NSP1 (from both SARS-CoV-2 and 1) was expressed the lowest, R124A and RK124AA mutants had intermediate expression levels, and KH164AA mutant was expressed the highest (Fig. 1C,D; Supplemental Fig. S1B).

The mRNAs of SARS-CoV-1 (Tanaka et al. 2012) and SARS-CoV-2 (Banerjee et al. 2020; Shi et al. 2020; Tidu et al. 2020; Mendez et al. 2021; ) have been reported to escape NSP1-mediated repression through interactions with NSP1 itself. To recapitulate this process, we added SARS-CoV-2 leader to our Renilla luciferase reporter (CoV-2-RL). Under this condition, the expression of CoV-2-RL mRNA was not repressed (Fig. 1E). Moreover, low doses of WT NSP1 stimulated the expression of CoV-2-RL at a level of 2.5- to threefold. In SARS-CoV-1, the R124A NSP1 mutant has been reported to be defective in its binding to the viral leader (Tanaka et al. 2012). Therefore, we tested whether SARS-CoV-2 RK124AA and R124A mutants were still able to repress CoV-2-RL. Strikingly, both RK124AA and R124A mutants continued to repress CoV-2-RL. We observed similar behavior for SARS-CoV-1 NSP1 and its mutants (Fig. 1F).

**FIGURE 2.**
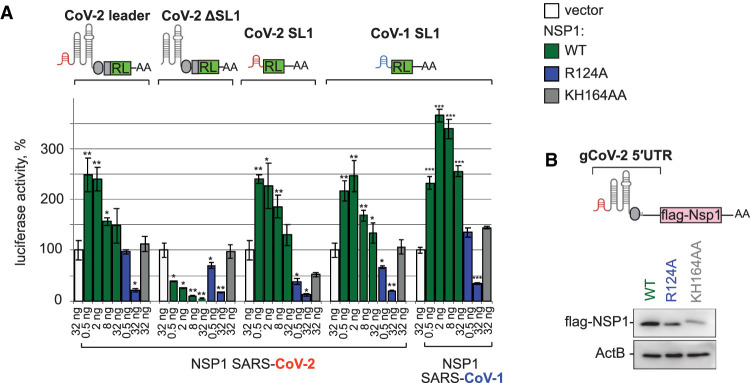
SARS-COV-2 leader alleviates NSP1-mediated silencing via its stem–loop 1 (SL1) structure and enhances NSP1 expression. (*A*) SL1 in SARS-COV-2 leader is both necessary and sufficient to escape NSP1-mediated repression. HEK293T cells were cotransfected with the indicated SARS-COV-2 leader reporters and NSP1-encoding constructs, either WT or indicated mutants. CoV-2-RL reporter is the same as in Figure 1D–E (contains a full-length leader, TRS [gray oval] and the beginning of viral ORF9 fused with RL coding sequence), CoV-2-ΔSL1-RL lacks stem–loop 1 (SL1), and CoV-2-SL1-RL and CoV-SL1-RL carry SL1 alone, originating from SARS-CoV-2 and SARS-CoV, correspondingly. The experiment was performed, and data presented as in Figure 1C. Values are shown as a percentage of luciferase produced in the presence of the vector for each reporter. Values represent means ± SD from at least three experiments. *P*-values are calculated and presented as in Figure 1. (*B*) NSP1 encoded by mRNA carrying the viral leader enhances its own expression. Constructs encoding SARS-CoV-2 NSP1 or its point mutants and carrying 5′UTR of genomic SARS-CoV-2 RNA (gCoV-2-flag-NSP1) were transfected in HEK293T cells, and cell lysates were analyzed by western blotting with anti-flag antibody. Beta-actin was used as a loading control.

We next decided to determine whether a specific region of the SARS-CoV-2 leader is responsible for alleviation of NSP1 silencing. To achieve this, we performed a deletion analysis of SARS-COV-2 leader in reporter assay. Given earlier reports on the role of stem–loop 1 (SL1) in the expression of SARS-CoV-1 (Tanaka et al. 2012) and SARS-CoV-2 (Banerjee et al. 2020; Shi et al. 2020; Tidu et al. 2020), we generated reporters in which SL1 is deleted (CoV-2 ΔSL1-RL), or which contain only the SL1 region (CoV-2 SL1-RL, CoV-1 SL1-RL). This analysis showed that SL1 is both necessary and sufficient to escape NSP1-mediated repression (Fig. 2A). Consistently, when we generated NSP1-encoding constructs carrying native SARS-CoV-2 leader, we observed that WT NSP1 was expressed at higher levels than its point mutants (Fig. 2B). These results suggest that viral-encoded NSP1 enhances its own expression and that of other viral proteins, while inhibiting the expression of host mRNAs.

### Three cytosine residues in SL1 are necessary for its derepressor function

**FIGURE 3.**
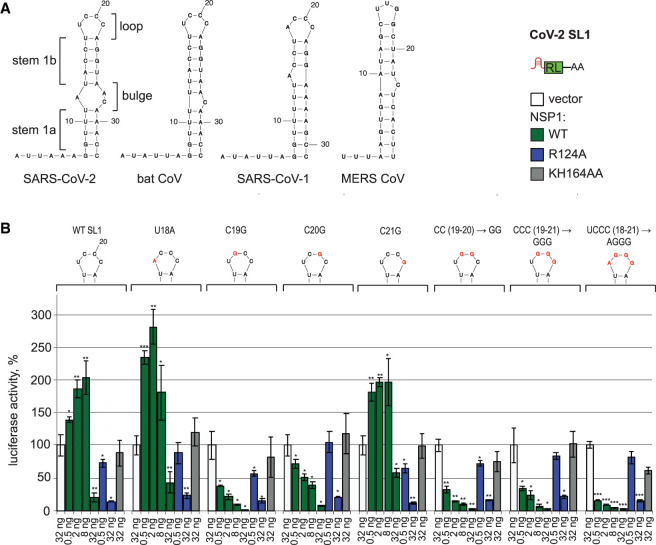
Positions C19 and C20 in the loop of SARS-CoV-2 SL1 are required to escape NSP1-mediated repression. (*A*) Structures of SARS-CoV-2, bat CoV, SARS-CoV, and MERS-CoV SL1, predicted by mfold (Zuker 2003). The elements of SL1 are labeled: loop, two sections of stem 1 (stem1a and stem 1b), separated by bulge. (*B*) Mutagenesis of the loop shows the requirement of C19 and C20 for the derepressor function of SL1 in reporter assay. HEK293T cells were cotransfected with SL1-RL reporter or indicated SL1 mutants and the constructs encoding SARS-CoV-2 NSP1, either WT or indicated mutants. The introduced mutations (18U > A, 19C > G, 20C > G, 21C > G, [19C > G; 20C > G], [19C > G; 20C > G; 21C > G], [18U > A; 19C > G; 20C > G; 21C > G]) and resulting sequences of the loop are shown *above* the plots. The mutated residues are shown in red. The results are presented as in Figure 1C. Values are shown as a percentage of luciferase produced in the presence of the vector for each reporter. Values represent means ± SD from at least three experiments. *P*-values are calculated and presented as in Figure 1.

In a next step, we mapped the residues within SL1 which are required for its function as a derepressor. To this end, we carried out extensive mutagenesis of SL1 and tested how specific mutations affected the expression of the SL1-RL reporter in the presence of NSP1. SL1 is highly conserved between SARS-CoV-2, bat CoV and SARS-CoV-1, while MERS-CoV SL1 shows less conservation (Fig. 3A). SARS-CoV-2 SL1 consists of two 10 bp-long double helices (stem 1a and 1b), with a bulge in between, and a 4 nt-long loop. Given the conservation of the loop sequence (U/ACCC), we first mutated the residues within it and tested how these mutations affected the ability of SL1 to escape NSP1-mediated repression in the luciferase reporter assay. The 18U > A (i.e., at the position 18 of SL1 a U was changed to an A) and 21C > G mutants were functional. But mutations of 19C and 20C, individually or in combination (19C > G, 20C > G, [19C > G; 20C > G], [19C > G; 20C > G; 21C > G], [18U > A; 19C > G; 20C > G; 21C > G]), disrupted the derepressor function of SL1 (Fig. 3B).

**FIGURE 4.**
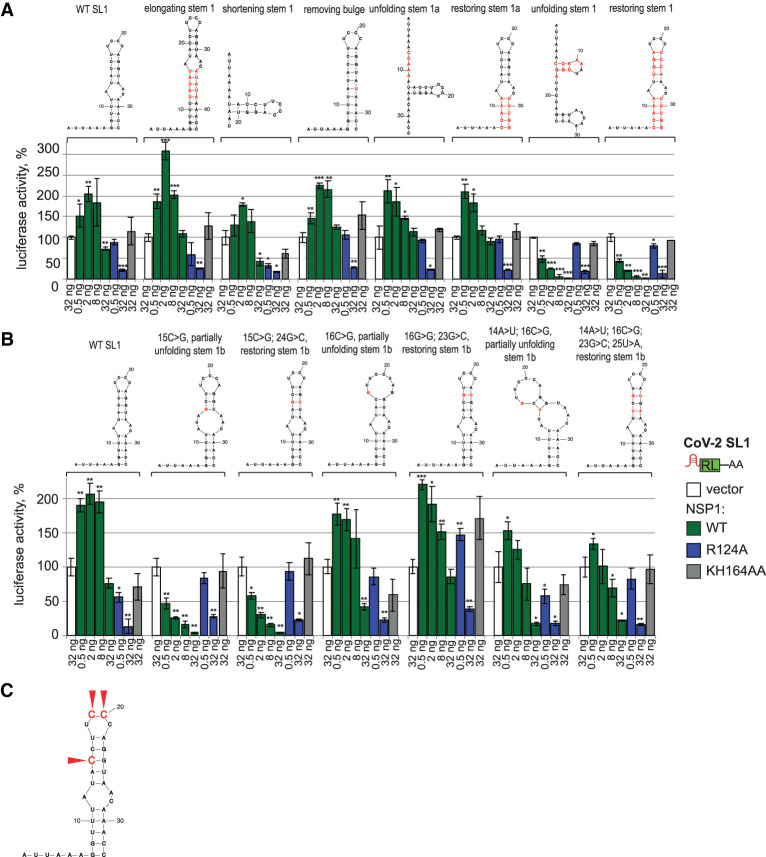
Position C15 in the stem of SARS-CoV-2 SL1 is necessary to escape NSP1-mediated repression. (*A*) Sequence of stem 1b (see Fig. 2A) is important for derepressor function of SL1. The role of mutations, disrupting folding or modifying parameters of the stem and bulge in SL1, was tested in the NSP1-reporter assay, as described in Figure 3B. Values are shown as a percentage of luciferase produced in the presence of the vector for each reporter. Values represent means ± SD from at least three experiments. *P*-values are calculated as in Figure 1. Mutations and resulting predicted structures (mfold, Zuker 2003) are shown *above* the plots, with mutated residues in red: 7_11del GGUUUinsCCAAA (i.e., replacement of nucleotides 7 to 11 [GGUUU] by CCAAA) unfolding stem 1a, [17_11delGGUUUinsCCAAA; 29_33delAAACCinsUUUGG] restoring stem 1a, [7_11del GGUUUinsCCAAA; 14_17delACCUinsUGGA] unfolding the stem 1, [7_11del GGUUUinsCCAAA; 14_17delACCUinsUGGA; 22_25delAGGUinsUCCA; 29_33delAAACCinsUUUGG] restoring stem 1, [12_16insUAUAU; 34_38AUAUA] elongating stem 1 by 5 bp, [7_11delCCAAA; 29-33del UUUGG] shortening stem 1 by 5 bp, 27_28delACinsU removing bulge. (*B*) Position 15C in stem 1b is required for SL1 function. Point mutations unfolding stem 1b, as well as compensatory mutations restoring folding, were introduced into SL1-RL reporter and tested in NSP1-mediated repression assay, as described in *A*. Mutations introduced in stem 1b: 15C > G unfolding; [15C > G; 24G > C] restoring folding; 16C > G unfolding; [16C > G; 23G > C] restoring folding; [14A > U; 16C > G] unfolding; [14A > U; 16C > G; 23G > C; 25U > A] restoring folding. (*C*) Three cytosine residues in SARS-CoV-2 SL1, one in stem 1b and two in the loop, are crucial to escape NSP1-mediate repression. Functional residues are marked with red arrows. The data are based on reporter assays shown in Figures 3B, 4A,B.

We next tested whether features of the stem such as its length or the presence of the bulge affect the function of SL1. We found that either extending or shortening the stem by 5 bp preserved part of the SL1 activity. NSP1 was not able to repress these reporters, although the overall efficiency of the expression of mutated SL1-RL and the degree of up-regulation by NSP1 were on average lower than for WT SL1-RL (Fig. 4A). Similar results were observed for SL1 without the bulge (Fig. 4A).

Our next question was whether the stem simply functions as a secondary structure that is required to present the loop in the right orientation, or whether the stem's sequence is also functionally important. To test this, first we mutated one of the strands to unfold the stem, and then introduced compensatory mutations in the second strand to restore the secondary structure. Unfolding the first part of the stem, located prior to the bulge (Fig. 4A, unfolding stem 1a) preserved most SL1 activity, consistent with our results from the shortening experiments. However, unfolding the entire stem fully abrogated the derepression activity of SL1 (Fig. 4A, unfolding stem 1). Introducing compensatory mutations that restored the stem, did not bring back its activity. These results suggest that the structure of the stem per se is not sufficient for the derepressor function of SL1; instead, the sequence of the stem contributes to its function.

To explore which residues in the stem are important, we introduced point mutations. Because mutations of stem 1a were tolerated (Fig. 4A), we mutated individual residues in stem 1b, adjacent to the loop. While mutations 16C > G and [14A > T; 16C > G] preserved much of the function, a mutation at position 15 fully abrogated SL1 function (15C > G, Fig. 4B). Importantly, restoring the complementarity of the strands by introducing a compensatory mutation in the second strand of stem 1b did not restore stem activity ([15G > G; 24G > C], Fig. 4B). This suggests that the specific sequence at this position is required for SL1 function.

To summarize, our analyses (Figs. 3, 4) identified three cytosines in SL1 which are required to provide for high expression of viral reporter: 15C, 19C, and 20C (red arrows, Fig. 4C).

### Targeting viral SL1 with antisense oligo (ASO)

**FIGURE 5.**
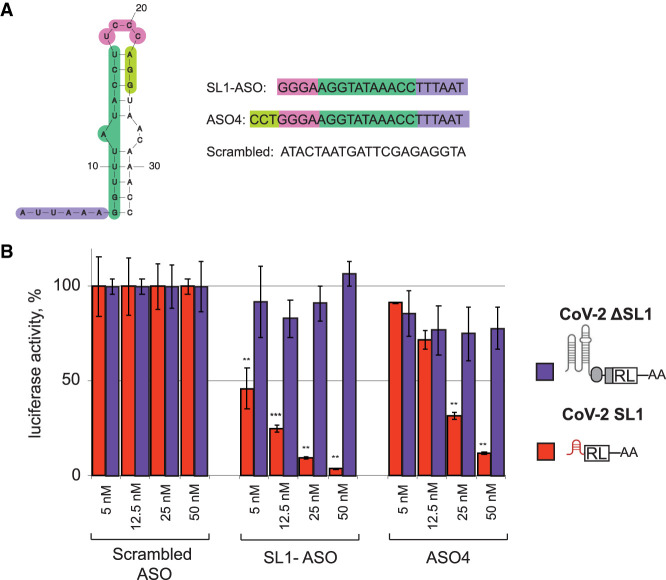
ASO targeting SL1 efficiently and specifically down-regulates viral RNA. (*A*) Sequences of ASOs used in the experiment. The sequence of ASO4 is from the study by Vora et al. (2021). (*B*) SL1-ASO down-regulates CoV-2 SL1-RL in a wide range of concentrations. HEK293T cells were transfected with the indicated SARS-COV-2 reporters and increasing amounts of ASOs targeting SL1. Scrambled ASO was used as negative control. CoV-2-SL1-RL and CoV-2-ΔSL1-RL reporters are as described in Figure 2A. Values are shown as a percentage of luciferase produced in the presence of the corresponding concentration of scrambled ASO for each reporter. Values represent means ± SD from at least three experiments. *P*-values ([*] <0.1, [**] <0.01, [***] <0.001), calculated with a two-sample *t*-test comparing scrambled and SL1-targeting ASO samples, are shown *above* the relevant bars.

Given the importance of SL1 for viral evasion, we decided to target it with an antisense oligo (ASO). We designed a perfect complement to the 5′ part of the stem/bulge region and the loop sequences (SL1-ASO, Fig. 5A). SL1-ASO represents a gapmer with the DNA core and flanks composed of locked nucleic acids (LNA) in phosphorothioate (PS) backbone. Gapmers are designed to support RNase-H1-mediated cleavage of the target RNA duplexed with the DNA core of a gapmer (for review, see Crooke et al. 2021). An ASO with a randomly rearranged SL1-ASO nucleotide sequence (Scrambled–ASO) was used as a negative control. We then tested how a range of ASO concentrations (5–50 nM) would affect expression of a luciferase reporter with or without SL1 (CoV-2 SL1-RL and CoV-2 ΔSL1-RL, correspondingly).

We observed that SL1-ASO specifically down-regulated SL1-RL (red bars, Fig. 5B), but not ΔSL1-RL (purple bars), with the efficiency of down-regulation from approximately twofold at 5 nM to ∼20-fold at 50 nM of SL1-ASO. A recent study by Vora et al. (2021) reported a similar ASO targeting SL1 (ASO4): unlike SL1-ASO, it represents a DNA/LNA mixmer, is longer by 3 nt (Fig. 5A), and was reported to have lower efficiency than SL1-ASO, decreasing expression of viral reporter by approximately twofold at 25–50 nM. To compare these two ASOs in the same experimental setup, we included ASO4 in our reporter assays. ASO4 was inefficient at lower concentrations (Fig. 5B, 5 and 12.5 nM), and lead to a three- to eightfold decrease in SL1-RL expression at higher concentrations (25 and 50 nM, correspondingly). Thus, our SL1-ASO design outperforms previously reported ASO4 by approximately threefold and is efficient at lower concentrations.

### NSP1 interacts with ribosomal proteins, mRNA export, and antiviral defense components

**FIGURE 6.**
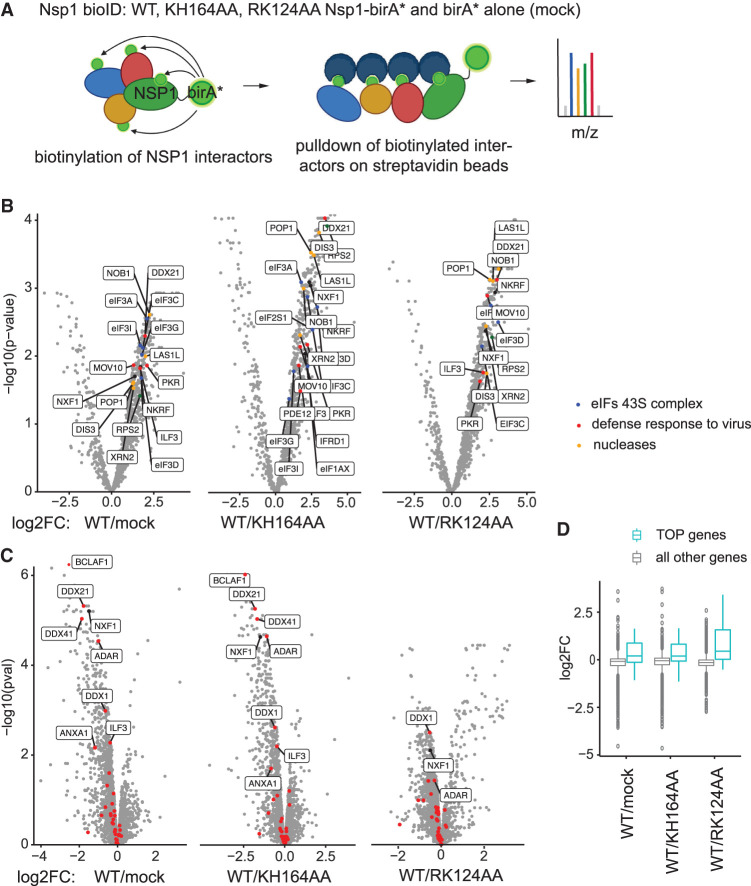
NSP1 interacts with ribosomal proteins, ribosome biogenesis factors and viral response factors. (*A*) Scheme for NSP1 BioID. NSP1 is fused with a promiscuous biotin ligase (birA), leading to labeling of proteins within a ∼10 nm radius. Labeled proteins are isolated on streptavidin beads and quantified by LC-MS/MS. Unfunctional mutants of NSP1 and biotin ligase alone (mock) serve as negative controls. (*B*) Volcano plots showing proteins enriched in SARS-CoV-2 NSP1-BioID. −log_10_ *P*-values (Y) are plotted against log_2_ fold change of LFQ (label-free quantification) values between NSP1-BioID and the indicated NSP1 mutant or BioID alone (mock) (X). Specific ribosomal protein (green), nuclear export factor 1 (NXF1, black), viral defense factors (referred to in the text and assigned to the GO category “defense response to virus,” red), and nucleases (orange) are shown. (*C*) Volcano plots showing changes in total proteome of 293T cells upon expression of SARS-CoV-2 NSP1. −log_10_ *P*-values (Y) are plotted against log_2_ fold change of LFQ (label-free quantification) values between NSP1 and the indicated NSP1 mutant or tag alone (mock) (X). (*D*) Boxplot showing changes in expression of proteins encoded by TOP genes (cyan) and all other genes (gray) upon expression of WT NSP1, compared with mock (WT/mock), KH164AA NSP1 mutant (WT/KH164AA), and RK124AA mutant (WT/RK124AA).

To characterize the functional interactome of NSP1, we applied BioID (Roux et al. 2012), which outperforms pulldown assays in catching transient interactors. This assay relies on fusing the protein of interest with a promiscuous biotin ligase (birA*, Fig. 6A). The ligase biotinylates any proteins in its close proximity (∼10 nm). These biotinylated proteins are subsequently purified on streptavidin beads and analyzed using mass spectrometry. We used this assay to identify the interactome of SARS-CoV-2 NSP1. As negative controls, we expressed biotin ligase alone (mock) or NSP1 mutants, KH164AA and RK124AA.

NSP1 has been reported to inhibit host translation by inserting its carboxy-terminal domain into the mRNA entry tunnel on the ribosomal 40S subunit (Schubert et al. 2020; Thoms et al. 2020; Lapointe et al. 2021). Consistent with these data, we detected interaction of WT NSP1 with PRS2/uS5 ribosomal protein situated near the mRNA entry tunnel (Fig. 6B, NSP1/mock, Supplemental Table S1). Other ribosomal proteins, located within the radius of birA* activity (∼10 nm) from the mRNA entry tunnel, were also detected among interactors. Other components of the 43S preinitiation complex—initiation factors eIF1A, eIF2, and eIF3—were also detected (Fig. 6B, blue). Importantly, the KH164AA NSP1 mutant, reported to be defective in interaction with the ribosome (Schubert et al. 2020; Thoms et al. 2020; Lapointe et al. 2021), failed to interact with ribosomal proteins and initiation factors (Fig. 6B, WT/KH164AA). The RK124AA NSP1 mutant was able to repress mRNA, although not as efficiently as WT NSP1 (Fig. 1A,B). Consistently, the RK124AA mutant interacted with ribosomal proteins and initiation factors weaker than WT NSP1 (Fig. 6B, WT/RK124AA). GO term enrichment analysis showed that interactors of WT NSP1 are linked with ribosomal terms (Supplemental Fig. S2).

Moreover, we found an enrichment of the nuclear export factor NXF1 in NSP1 BioID (Fig. 6B), in line with the recent data showing that SARS-CoV-2 disrupts mRNA export from the nucleus (Burke et al. 2021; Zhang et al. 2021). Additionally, the levels of NXF1 were down-regulated in cells expressing WT NSP1, when compared with mock or KH164AA (Fig. 6C). Besides changes in NXF1 levels, we observed that WT NSP1 up-regulated proteins, encoded by mRNAs with 5′ terminal oligopyrimidine (TOP) tracts (Fig. 6D, compare cyan and gray boxes). These data are consistent with the recent report that TOP mRNAs preferentially escape global suppression of translation by NSP1 (Rao et al. 2021).

Having established that our experiments identify known NSP1 binders and changes in total proteome, we also searched for possible novel interactors. Curiously, we found that WT NSP1 interacts with multiple components of the cellular antiviral defense system (Fig. 6B, red). The components we found include eukaryotic initiation factor 2 alpha (eIF2A, or eIF2S1) and eIF2A protein kinase R (PKR). PKR plays a protective role during viral infection: it is activated by double-stranded viral RNA, which leads to the phosphorylation of eIF2A and an inhibition of the synthesis of viral proteins (for review, see Gal-Ben-Ari et al. 2018). We identified further interactions with a number of other antiviral components, including: NKRF (NF-kB-repressing factor), which mediates transcriptional repression of NK-kappa-B responsive genes (Feng et al. 2002); IFRD1 (interferon-related developmental regulator 1), which suppresses NF-kB activation (Tummers et al. 2015); PDE12 (Phosphodiesterase 12), an enzyme that negatively regulates innate immunity (Wood et al. 2015); a number of helicases involved in IFN induction, including DDX21 and MOV10 (Zhang et al. 2011; Cuevas et al. 2016); ILF3 (Interleukin enhancer-binding factor 3), required for translation of antiviral cytokine IFNB1 and a subset of INF-stimulated genes (Wathelet et al. 2007). These and additional interactions uncovered in our assay suggest that NSP1 may modulate antiviral pathways via direct interactions with their components. Indeed, levels of some interactors, including DDX21, DDX1, and ILF3, were down-regulated upon NSP1 expression, compared with the mock and KH164AA-expressing cells (Fig. 6C). The RK124AA mutant, that retains part of NSP1 repressive potential, showed fewer differences with WT NSP1 with respect to changing the levels of these binders (Fig. 6C, WT/RK124AA). Curiously, we also observed that WT NSP1 down-regulated other components of the antiviral defense pathway, including ADAR, involved in coronavirus genome editing (Di Giorgio et al. 2020); Bcl-2-associated transcription factor 1 (BCLAF1), that induces proinflammatory cytokines IL-6 and IL-8 (Shao et al. 2016); Annexin A1 (ANXA1), that up-regulates cytoplasmic RNA sensor RIG-I and thereby stimulates IFNβ production (Yap et al. 2020).

NSP1 regulates both translation and stability, and the RK124AA NSP1 mutant from SARS-CoV-1 (Lokugamage et al. 2012) and SARS-CoV-2 (Mendez et al. 2021) was reported to be defective in mRNA destabilization. It has been speculated, that NSP1 might recruit a cellular nuclease in a manner dependent on intact R124 and K125 residues. We therefore looked for nucleases enriched among WT NSP1 interactors, compared with the RK124AA mutant (Fig. 6B, orange). Five nucleases have been identified: POP1, a component of ribonuclease P that generates mature tRNA by cleaving their 5′-ends (Lygerou et al. 1996); NOB1, an endonuclease required for processing of pre-rRNA precursor (Fatica et al. 2003); ribosomal biogenesis protein LAS1L (Castle et al. 2010); DIS3, a component of the RNA exosome complex which possesses both 3′ → 5′ exoribonuclease and endonuclease activity (Lebreton et al. 2008); and 5′ → 3′ exoribonuclease XRN2 (Skourti-Stathaki et al. 2011).

## DISCUSSION

To date, the most prominent preventive approach to combat the impact of SARS-CoV-2 has revolved around the development of vaccines that target the viral spike protein. However, vaccination does not fully stop the propagation of disease, and new variants of SARS-CoV-2 may emerge which prove resistant to existing vaccines. This heightens the need for the development of drugs that target the core machinery of SARS-CoV-2 and can be used to treat infected individuals. NSP1 seems to be an ideal candidate as a target: it is conserved in beta-coronaviruses; it plays a crucial role in both down-regulating the expression of host genes and promoting its own propagation; and it subdues the antiviral arsenal of infected cells. Assessing NSP1's potential as a target will require the type of detailed mechanistic understanding of its functions that we present here.

The mechanism we describe offers an explanation for the way SARS-CoV-2 down-regulates host mRNAs while concomitantly enhancing its own expression (Fig. 1; Supplemental Fig. S3). The first step in this process is the early expression of viral protein NSP1. NSP1 blocks host translation by inserting its carboxy-terminal domain into the mRNA entry tunnel on the ribosomal 40S subunit (Schubert et al. 2020; Thoms et al. 2020; Lapointe et al. 2021). Consistently, we detected interaction of WT NSP1, but not its nonfunctional mutant KH164AA, with ribosomal proteins and other components of the 43S preinitiation complex (Fig. 6B). Interestingly, a similar mechanism involving blocking of the mRNA entry tunnel has been previously reported for other translational repressors, SERBP1 (Brown et al. 2018) and Stm1 (Ben-Shem et al. 2011).

Moreover, NSP1 interacts with the components of antiviral machinery in cells (Fig. 6B) in a way that suggests that it may directly hijack antiviral pathways, beyond repressing the translation of host proteins by binding to the ribosome. In other cases, viral RNA is typically sensed by RNA helicases in infected cells, resulting in the activation of the transcription factors ATF2/c-Jun, IRF3/IRF7, and NF-kB (Maniatis et al. 1998). These in turn induce the production of cytokines, including members of the IFN family, which go on to bind to their cognate receptors and trigger a second wave of signaling. These two waves up-regulate genes that inhibit viral replication. By interacting with components of these pathways, NSP1 may prevent the full induction of IFNs as another mechanism that facilitates viral propagation. Indeed, we observed down-regulation of some of these factors upon NSP1 expression (Fig. 6C). Moreover, NSP1 has been speculated to destabilize mRNA (Lokugamage et al. 2012; Mendez et al. 2021) by recruiting a cellular nuclease in a manner dependent on R124 and K125 residues. Curiously, we have identified five nucleases among the NSP1 interactors lost in the RK124AA mutant (Fig. 6B), providing an important resource for future exploratory studies on SARS-CoV-2. Further experiments in the context of viral infection will be required to investigate how expression of other viral proteins may influence NSP1 interactome.

Previous findings that SARS-CoV-2 disrupts the production of cellular proteins left unanswered questions. To propagate, viruses must synthesize their own proteins, and this depends on the very machinery that appeared to be suppressed. Our work presents a mechanism by which SARS-CoV-2 ensures the translation of its own RNA, which depends on NSP1. We show that the stem loop SL1 within the viral leader sequence is both necessary and sufficient to escape NSP1-mediated repression (Fig. 2A); this confirms findings by Banerjee et al. (2020) and Tidu et al. (2020).

Interestingly, Schubert et al. (2020) failed to detect viral evasion in their reporter assays and suggested that virus may use a different strategy. They proposed that viral transcripts might have a higher translation efficiency to begin with, which would give them a kinetic advantage in translation over cellular transcripts. Our data suggest a more likely explanation for the discrepancy in the two models. The viral leader used in Schubert's study (2020) carries five additional nucleotides at the 5′ end introduced during cloning (according to the Materials and Methods section of the manuscript). Such additional nucleotides abrogated an evasion mechanism in both our (data not shown) and other studies (Banerjee et al. 2020).

Interaction studies conducted with SARS-CoV-1 and SARS-CoV-2 NSP1 suggested that it can be bound by SL1 (Tanaka et al. 2012; Shi et al. 2020; Tidu et al. 2020). This likely causes NSP1 to be expelled from the mRNA entry tunnel. Curiously, Mendez et al. (2021) detected interaction of NSP1 with both viral and cellular reporter mRNAs, while the earlier study of Tanaka et al. (2012) on SARS-CoV-1 reports that a viral leader is required for such an interaction. Further structural studies will be required to resolve this discrepancy and uncover the specific mechanism behind the structural rearrangements of NSP1 on the ribosome that potentially occur upon its binding to SL1. Importantly, our analysis pinpoints specific residues within SL1 (three cytosine residues at the positions 15, 19, and 20) and NSP1 (R124) which are required for viral evasion and are likely involved in SL1/NSP1 interactions.

Our experiments revealed a dose-dependent response of viral reporters to NSP1. Specifically, at high doses of NSP1, viral reporters simply escaped silencing, but at low amounts, NSP1 actually stimulated their expression. We speculate that the enhancement of viral translation is caused by the global repression of host translation by NSP1. This generates a pool of translation factors and ribosomes that can now be co-opted by the virus. This principle is known; it has been established for other viruses which carry internal ribosome entry sites and make use of a similar hijacking mechanism (for review, see Lee et al. 2017). But this effect has not been previously reported for SARS-CoV-2 NSP1. The reason probably lies with the structure of prior studies. In some cases, they have not compared expression of viral reporters in the presence and in the absence of NSP1 (Banerjee et al. 2020). Other studies have observed a similar expression of viral reporters under both conditions (Shi et al. 2020; Mendez et al. 2021) or detected a weaker repression of viral reporters, compared with nonviral reporters (Tidu et al. 2020). The results of Shi et al. (2020) and Mendez et al. (2021) are consistent with our reporter assays in the presence of high levels of NSP1 and are easiest explained by high amounts of NSP1-encoding plasmid used in these studies. The most likely explanation for the effects observed by Tidu et al. (2020) is that this work was performed in rabbit reticulocyte lysates, which are typically treated with nucleases to eliminate endogenous mRNAs. This system therefore does not recapitulate a possible competition for a limited number of translation factors, which is characteristic for translation in vivo.

An intriguing question remains regarding the extent to which NSP1 up-regulates the expression of viral RNAs in the context of actual viral infection. Future in vivo experiments with viruses carrying mutant forms of NSP1 and SL1 will be required to address this question.

NSP1's and SL1's fundamental roles in viral infections make them highly interesting potential targets for drugs. Antisense oligos (ASOs) targeting RNA is a highly promising technology, with nine approved ASO-based drugs up to date (for review, see Crooke et al. 2021). While targeting secondary structures with ASO may be challenging, we identified SL1-ASO that binds SL1 and specifically down-regulates viral RNA by ∼10-fold at 25 nM concentration. For comparison, previously reported SARS-CoV-2-targeting ASOs down-regulated viral RNA by ∼1.5-fold at 25 nM (Vora et al. 2021) and 5 nM concentration (Zhu et al. 2021). Besides ASOs, screening for compounds that interfere with NSP1 and SL1 interactions is a promising strategy for drug development. The understanding we have gained of the mechanisms underlying its functions suggest three potential points of attack. The most obvious place to interfere is the site of the protein that interacts with the ribosome and blocks the mRNA entry tunnel. This is the defect observed in the KH164AA NSP1 mutant, which fails to interact with ribosome and is nonfunctional. Targeting K164 and H165 with small molecules therefore appear to be a promising strategy that would disrupt the pathogenicity of SARS-CoV-2. Another weak point that could be exploited is the mechanism which viral molecules use to evade NSP1 silencing: the structure that permits NSP1 to interact with the viral leader. There are two possible targets: the regions in either NSP1 or SL1 that permit and are required for this interaction. Our finding that the R124A mutant, but not WT NSP1, effectively represses the viral reporter points to a vulnerable spot on NSP1 that could be targeted by therapies. On the SL1 side, finding compounds that target three crucial cytosines (C15, C19, and C20), alone or in combination, might hold a great potential for the development of novel SARS-CoV-2 therapies.

## MATERIALS AND METHODS

### Cell culture, transfections, and luciferase assay

Human HEK293T cells were grown in Dulbecco's modified Eagle's medium with GlutaMAX supplement (DMEM+ GlutaMAX, GIBCO) with 10% FBS. Transfections were done in 96-well plates with polyethylenimine (PEI) using a 1:3 ratio of DNA:PEI. In reporter experiments, HEK293T cells were transfected with 1–2 ng RL, 15 ng FL, and indicated amounts of NSP1-encoding constructs per well of a 96-well plate. The total amount of transfected DNA was topped up to 50 ng per well of a 96-well plate with the vector. Cells were lysed 24 h post transfection. Luciferase activities were measured with a homemade luciferase reporter assay system as described earlier (Mauri et al. 2016).

### DNA constructs and ASOs

3xflag-SARS-CoV-2 NSP1-encoding plasmid and pEBG-3xflag, used as a vector, have been described previously (Mendonsa et al. 2021). Analogous plasmid expressing SARS-CoV-1 NSP1 was generated using a similar strategy: CDS of NSP1 was PCR amplified, using SARS-CoV-1 cDNA as a template, and cloned between the SbfI and NotI sites of pEBG-3xflag. R124A, [R124A; K125A], and [K164A; H165A] mutations were introduced in SARS-CoV-2 and SARS-CoV-1 NSP1 CDS by site-directed mutagenesis. GST-SARS-CoV-2 NSP1-encoding plasmid was generated by cloning NSP1 CDS into SbfI-NotI-cut pEBG (Addgene #22227). RL reporter is similar to the previously described RL plasmid (Pillai et al. 2004) but lacks the last 8 nt in the CMV promoter, which were removed by site-directed mutagenesis. RL served as a backbone for cloning of Renilla reporters carrying SARS-CoV-2 leader, as well as its deletion and point mutants. SARS-CoV-2 leader with adjacent sequences (attaaaggtttataccttcccaggtaacaaaccaaccaactttcgatctcttgtagatctgttctctaaacgaacaaactaaaatgtctgataatggacccca) was generated by oligo annealing and cloned between SacI and NheI sites of RL, to produce CoV-2-RL. For CoV-2-ΔSL1-RL, the leader lacking the first 33 nt (aaccaactttcgatctcttgtagatctgttctctaaacgaacaaactaaa) was cloned upstream of RL. SL1-RL (or CoV-2-SL-RL) and CoV-1-SL1-RL contain the first 33 nt of SARS-CoV-2 (attaaaggtttataccttcccaggtaacaaacc) or the first 31 nt of SARS-CoV-2 leader (atattaggtttttacctacccaggaaaagcc), respectively. Mutations of SL1, indicated in the figures, were introduced into the oligos used for cloning. CoV-2-FL reporter is analogous to CoV-2-RL, with *Renilla* CDS substituted for firefly CDS. To clone gCoV-2-flag-NSP1 plasmids, CDS of NSP1 and its point mutants were PCR amplified and cloned between NheI and NotI sites of RL, to substitute RL CDS. At the next step, the SARS-CoV-2 genomic leader was PCR amplified using SARS-CoV-2 cDNA as a template (5′ end: attaaaggtttataccttcccagg; 3′ end: cttacctttcggtcacacccggac) and cloned it between SacI and NheI of NSP1 plasmids. For BioID constructs, BioID CDS was PCR amplified from pcDNA 3.1-BioID (Roux et al. 2012) and cloned between BstXI and NotI of pEBG-sic (Chekulaeva et al. 2011), to produce pEBG-BioID. pEBG-BioID was used as a mock control in BioID experiments and as a backbone for cloning NSP1-myc-BioID constructs. For that, CDS of NSP1 or its point mutants were PCR amplified and cloned between NheI and SmaI sites of pEBG-BioID.

ASOs (Sigma) are composed of DNA and locked nucleic acids (LNA) nucleotides in phosphorothioate (PS) backbone. LNAs are denoted with [+].

  SL1-ASO: [+G][+G][+G]AAGGTATAAACCTTT[+A][+A][+T];
  Scrambled: [+A][+T][+A]CTAATGATTCGAGAG[+G][+T][+A];
  ASO4: C[+C]T[+G]G[+G]A[+A]G[+G]T[+A]T[+A]A[+A]C[+C] T[+T]T[+A]A[+T] (Vora et al. 2021).

### Western blotting and BioID

For western blotting, 20 µL of total cell lysate from reporter assay was separated on a 10% SDS-PAGE, and proteins were transferred to the PVDF membrane. The membrane was probed with the following primary antibodies: mouse anti-flag antibody 1:2000 (F1804 Sigma), mouse anti-beta-actin 1:5000 (A2228 Sigma).

For BioID experiments, HEK293T cells were transfected with constructs encoding NSP1-BioID, NSP1-KH164AA-BioID, NSP1-RK124AA- BioID, or BioID alone. Transfections were done in quadruplicates, using 10 µg of plasmid and 30 µg of polyethylenimine (PEI) per 10 cm dish with 3 × 10^6^ cells plated a day before transfection. Cell culture medium was supplemented with 50 µM biotin. Cells were lyzed 24 h post-transfection, and BioID was performed as previously described (Roux et al. 2018). In short, cells were lyzed in 8 M urea, 50 mM Tris-HCl pH 7.4, 1× protease inhibitors Complete EDTA-free, 1 mM DTT. Lysates were supplemented with Triton-X100 to a final concentration of 1%, sonicated, diluted fivefold with lysis buffer and clarified. Biotinylated proteins were isolated by incubation with 100 µL of Streptavidin Dynabeads (Thermo 65001) at 4°C with rotation overnight. Proteins were eluted 2× with 25 µL of elution buffer (5% SDS, 50 mM Tris-HCl pH 7.4) and used for mass spectrometry analysis.

### Mass spectrometry: in solution digestion and LC-MS/MS analysis

The BioID IP eluates (10 µL each) were diluted in ultra-pure water whereas cell lysates, each corresponding to 20 µg of protein, were diluted in 50 mM ammonium bicarbonate (NH_4_HCO_3_) buffer, pH 7.8 to a final volume of 100 µL. All samples were reduced with 10 mM DTT at 56°C for 30 min, and subsequently alkylated with 30 mM IAA at room temperature for 30 min in the dark. Next, the samples were subjected to ethanol (EtOH) precipitation followed by in-solution protein digestion. Briefly, each sample was diluted 10-fold with ice-cold EtOH in 1:10 (v/v) ratio, vortexed and incubated at −40°C for 60 min followed by centrifugation in a precooled (4°C) centrifuge at 20,000*g* for 30 min. The obtained pellet was washed with 100 µL of ice-cold acetone, briefly vortexed and centrifuged as mentioned above for 5 min. The supernatant was discarded, and the pellet was dried under laminar flow hood and resolubilized in 60 µL of digestion buffer comprising: 0.2 M GuHCl, 2 mM CaCl_2_, 50 mM NH_4_HCO_3_, pH 7.8. 100 ng of Trypsin Gold and 1 µg of Trypsin Sequencing Grade (both Promega) were added to BioID eluates and total cell lysates, respectively, and subjected to proteolysis at 37°C for 16 h. Lastly, all samples were acidified with 10% TFA to pH <3.0, and an aliquot of each digest, that is, 10% of BioID and 5% of total cell lysate, was quality controlled as described previously (Burkhart et al. 2012).

For LC-MS/MS analysis*,* 30% of BioID eluates and 10% of total cell lysates digests were analyzed using an Ultimate 3000 nano RSLC system coupled to Orbitrap Lumos (both Thermo Scientific). Peptides were preconcentrated on a 100 µm × 2 cm C18 trapping column for 5 min using 0.1% TFA with a flow rate of 20 µL/min followed by separation on a 75 µm × 50 cm C18 main column (both Acclaim PepMap Nanoviper, Thermo Scientific) with a 60 min (BioID samples) or 120 min (cell lysate samples) LC gradient ranging from 3%–35% of B (84% ACN in 0.1% FA) at a flow rate of 250 nL/min. The Orbitrap Lumos was operated in data-dependent acquisition mode, and MS survey scans were acquired from m/z 300 to 1500 at a resolution of 120,000 using the polysiloxane ion at m/z 445.12002 as lock mass (Olsen et al. 2005). For MS1 scans, the automatic gain control (AGC) target value was set to 2 × 10^5^ with a maximum injection time (IT) of 50 msec. MS2 spectra were acquired in the linear ion trap (rapid scan mode) after higher-energy collisional dissociation with a normalized collision energy of 30%, an AGC target value of 2 × 10^3^, and a maximum IT of 300 msec, by utilizing a maximal duty cycle of 3 sec, prioritizing the most intense ions and injecting ions for all available parallelizable time. Selected precursor ions were isolated using quadrupole with a 1.2 m/z window taking into account a dynamic exclusion of 30 sec.

For data analysis, all MS raw data were processed with Proteome Discoverer software 2.3.0.523 (Thermo Scientific) and searched in a target/decoy fashion against a concatenated version of the human Uniprot database (downloaded on November 2019, 20300 target sequences); NSP1 from SARS-CoV-2 and BioID SEQUEST-HT algorithm. The search parameters were precursor and fragment ion tolerances of 10 ppm and 0.5 KDa for MS and MS/MS, respectively. Trypsin was set as enzyme with a maximum of two missed cleavages. Carbamidomethylation of Cys as fixed modification and oxidation of Met was selected as dynamic modification. The false discovery rate was set to 0.01 for both peptide and protein identifications using Percolator. A label-free quantification (LFQ) analysis was performed with four replicates for each condition for whole proteome analysis and for the pull-down experiment. Proteins identified with ≥2 unique peptides were used for differential expression analysis. Enrichment (log_2_ fold change) of proteins between pulldown fractions or lysate samples was calculated using a generalized linear model (R *limma* package, Ritchie et al. 2015) on imputed log_2_-transformed LFQ values. *P*-values, obtained from the empirical Bayes method implemented in *limma*, were adjusted for multiple testing using the FDR method.

## SUPPLEMENTAL MATERIAL

Supplemental material is available for this article.

## DATA DEPOSITION

The mass spectrometry proteomics data have been deposited to the ProteomeXchange Consortium via the PRIDE partner repository with the data set identifier PXD024480.

## Supplementary Material

Supplemental Material
