# Supplementary material for: The key features of SARS-CoV-2 leader and NSP1 required for viral escape of NSP1-mediated repression
Source: RNA. 2022 May;28(5):766–79. doi: 10.1261/rna.079086.121 (PMC9014875; doi:10.1261/rna.079086.121)
Supplement: Supplemental Material [file supp_28_5_766__DC1.html]

The key features of SARS-CoV-2 leader and NSP1 required for viral escape of NSP1-mediated repression — Supplemental Material 

# The key features of SARS-CoV-2 leader and NSP1 required for viral escape of NSP1-mediated repression

## Supplemental Material

- Supplemental\_Figures\_S1-S3.pdf
- Supplemental\_Table\_S1\_proteomics.xlsx
