# Supplementary material for: The key features of SARS-CoV-2 leader and NSP1 required for viral escape of NSP1-mediated repression
Source: RNA. 2022 May;28(5):766–79. doi: 10.1261/rna.079086.121 (PMC9014875; doi:10.1261/rna.079086.121)
Supplement: Supplemental Material [file supp_079086.121_Supplemental_Figures_S1-S3.pdf]

A

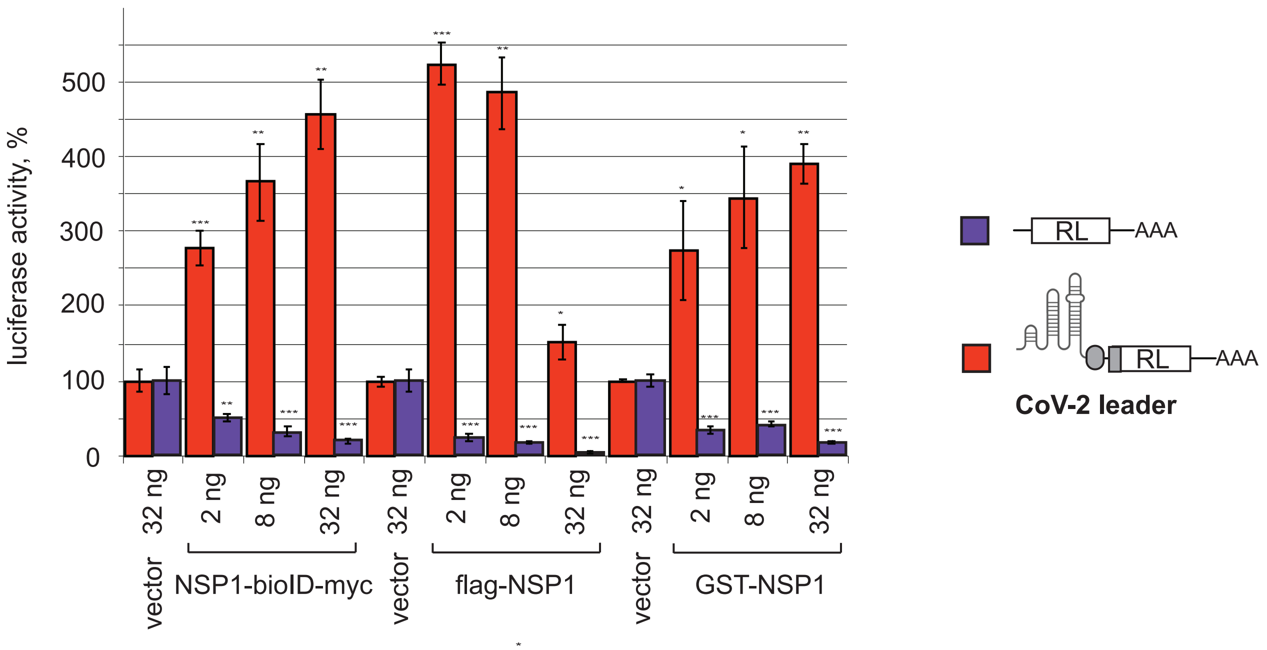

B

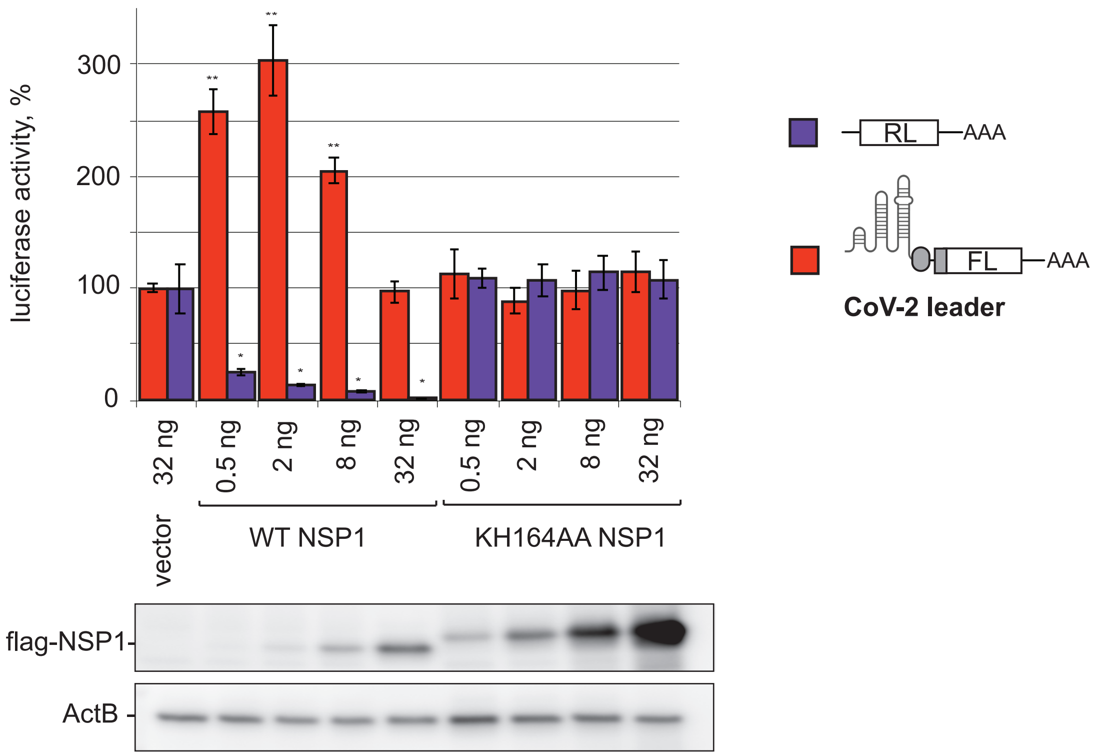

Figure S1. SARS-COV-2 leader enables virus to escape repression by NSP1 protein. (**A**) NSP1-mediated repression and viral evasion is observed for both N- and C-terminally tagged NSP1. HEK293T cells were co-transfected with Renilla luciferase plasmid, with or without SARS-CoV2 leader (CoV-2-RL: red bars, RL: purple bars), and increasing amounts of plasmids, encoding C-terminally (NSP1-bioID-myc) or N-terminally tagged SARS-CoV-2 NSP1 (flag-NSP1, GST-NSP1). As negative controls, vectors encoding tags alone instead of NSP1 were used. Values are presented as a percentage of luciferase produced in the presence of vector for each reporter. Values represent means +/-SD from at least 3 experiments. P-values are calculated and presented as in **Figure 1**. (**B**) KH164AA NSP1 is nonfunctional in repression in a wide range of concentrations and serves as an effective negative control for WT NSP1. HEK293T cells were co-transfected with Renilla luciferase plasmid (RL: purple bars), firefly luciferase plasmid with SARS-CoV2 leader (CoV-2-FL: red bars), and increasing amounts of plasmids, encoding flag-tagged SARS-CoV-2 NSP1, WT or KH164AA mutant. As negative control, vector encoding flag alone instead of flag-NSP1 plasmid was used. Values are shown as a percentage of luciferase produced in the presence of vector for each reporter. Values represent means +/-SD from at least 3 experiments. P-values are calculated and presented as in **Figure 1.** Expression of flag-NSP1 fusion protein, WT and KH164AA mutant, was estimated by western blotting with anti-flag antibodies and shown below the reporter assay. ActB was used a loading control.

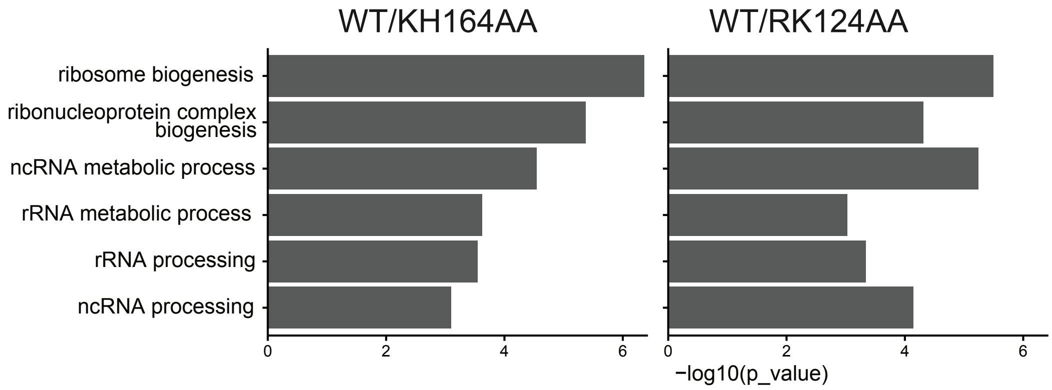

Figure S2. Gene Ontology (GO) terms enriched among WT NSP1 interactors compared with the indicated mutant. Proteins with significant (p.adjusted < 0.05) enrichment in bio-ID of WT NSP1 compared with mutant NSP1 (log2FC WT/mutant > 1) were used for GO analysis using gprofiler2. Proteins detected across all bio-ID samples were used as custom background for GO enrichment analysis. After removing enriched GO terms with less then 5 or more than 1000 annotated genes, the terms from the "biological processes" domain were retained for plots.

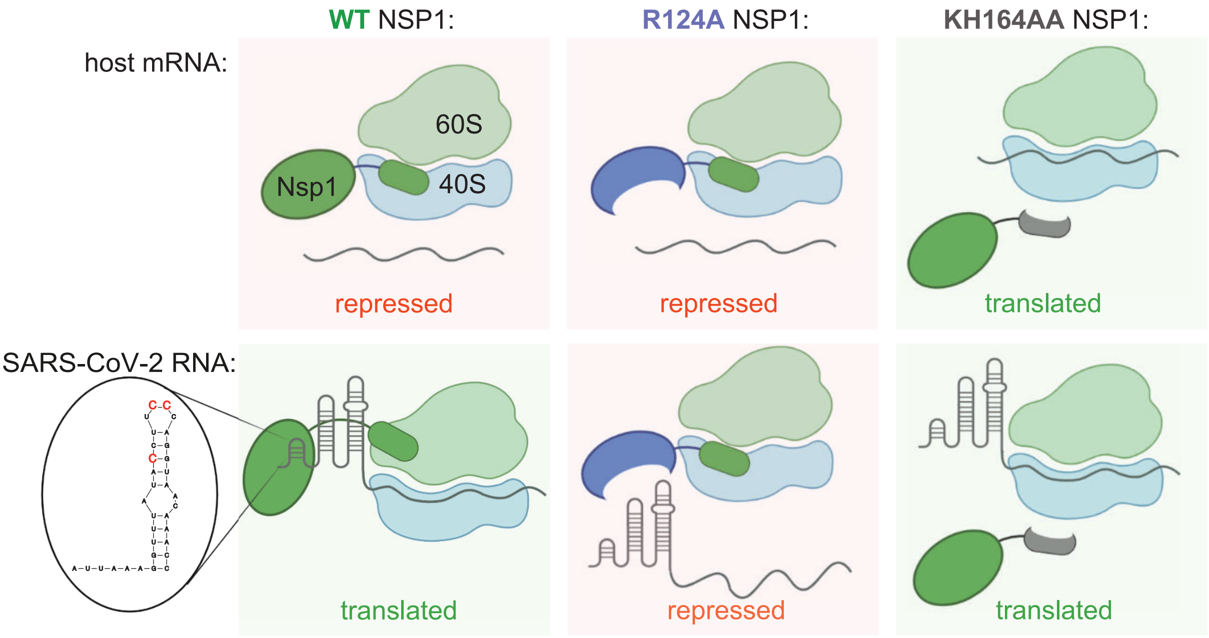

Figure S3. Speculative model for NSP1 function in repression of host mRNA and activation of SARS-CoV-2 expression. WT NSP1 protein represses translation of host mRNA via blocking the ribosome entry tunnel. SARS-CoV-2 genomic and subgenomic RNAs escape repression by NSP1 due to SL1 in their leader, interacting with NSP1. This way viral RNA highjacks host ribosomes without competing for limiting eIFs in infected cells. In particular, positions C15, C19 and C20 are required for alleviation of NSP1 silencing. Positions K164H165 in NSP1 are required for interaction with ribosome, therefore KH164AA mutant of NSP1 does not repress either host or SARS-CoV-2 mRNA. Position R124 is required for interaction with SL1, therefore NSP1 R124A mutant represses both host and viral mRNA.
